# Investigation of the role of GEM in systemic lupus erythematosus through multi-omics joint analysis

**DOI:** 10.3389/fimmu.2025.1569605

**Published:** 2025-04-09

**Authors:** Ruofei Chen, Xiao Zhang, Yifang Shang, Huaixuan Zhang, Xiaolei Li, Hanren Dai, Zongwen Shuai

**Affiliations:** ^1^ Department of Rheumatology and Immunology, the First Affiliated Hospital of Anhui Medical University, Hefei, China; ^2^ First College of Clinical Medicine, Shandong University of Traditional Chinese Medicine, Jinan, China; ^3^ Department of Critical Care Medicine, the Second Affiliated Hospital of Anhui Medical University and Institute of Clinical Pharmacology, The MOE Key Laboratory of Anti-inflammatory and Immune Medicine, Anhui Medical University, Hefei, China; ^4^ School of Pharmacy, Inflammation and Immune Mediated Diseases Laboratory of Anhui Province, Anhui Medical University, Hefei, China

**Keywords:** SLE, GEM, fibroblast, immunology, therapeutic target

## Abstract

**Background:**

Systemic lupus erythematosus (SLE) is a persistent autoimmune disorder marked by dysregulation of the immune system, resulting in extensive tissue inflammation and subsequent damage. Fibroblasts are essential contributors to the pathogenesis of SLE, particularly in driving the progression of tissue fibrosis and inflammation. Recent research has proposed that the GEM gene may regulate fibroblast activity in SLE. However, the precise molecular mechanisms through which GEM modulates fibroblast functions in the context of SLE are yet to be fully elucidated. Gaining insight into these mechanisms is crucial for uncovering potential therapeutic targets aimed at addressing fibrosis and inflammation associated with SLE.

**Methods:**

Single-cell RNA sequencing was integrated with cell-based assays, such as quantitative reverse transcription PCR (qRT-PCR) and functional cellular experiments, to investigate the underlying mechanisms. The regulatory mechanisms of GEM in fibroblasts were analyzed through functional cell assays.

**Results:**

Differential gene expression in fibroblast subpopulations was identified through single-cell RNA sequencing, with GEM emerging as a key gene implicated in these alterations. Trajectory analysis indicated that GEM expression correlated with fibroblast proliferation and migration. Subsequent experiments confirmed that GEM regulates fibroblast viability and influences SLE disease progression through modulation of cell proliferation, migration, and apoptosis.

**Conclusions:**

GEM is highly differentially expressed in fibroblast subpopulations within SLE, and its altered expression impacts fibroblast proliferation and migration. GEM may regulate fibroblast activity and apoptosis, potentially contributing to the progression of SLE.

## 1 Introduction

Systemic lupus erythematosus (SLE) is a persistent autoimmune disorder marked by extensive inflammation and tissue damage, which impacts various organ systems such as the skin, kidneys, heart, and nervous system (1, 2). Epidemiologically, SLE predominantly affects women, with a higher prevalence observed in individuals of African, Hispanic, and Asian descent (3). The disease often presents during the reproductive years, with peak onset between the ages of 15 and 45 (4). The global incidence of SLE varies by region, but it is generally estimated to affect approximately 20-150 per 100,000 people, depending on the population studied (5). Pathologically, SLE is characterized by immune system dysregulation, resulting in the generation of autoantibodies that attack the body’s own tissues, thereby inducing widespread inflammation (6, 7). The underlying mechanisms of SLE involve complex interactions between genetic susceptibility, environmental triggers, and immune system dysfunction (8). However, the precise molecular mechanisms driving disease onset and progression remain poorly understood, presenting a significant challenge in developing targeted therapies (9). Despite advancements in the understanding of SLE, the treatment options remain limited, with corticosteroids and immunosuppressive drugs being the mainstay therapies (10). These treatments aim to manage symptoms and prevent flare-ups but are associated with significant side effects and do not address the root causes of the disease. The lack of effective disease-modifying treatments and the incomplete understanding of the molecular underpinnings of SLE highlight the critical need for ongoing research to discover new therapeutic targets and strategies aimed at enhancing patient outcomes and quality of life.

GEM (GTPase-activating protein for cell migration and invasion), a member of the dynamin GTPase family, is crucial for regulating a variety of cellular processes, including vesicle trafficking, signal transduction, and cytoskeletal dynamics (11). Recent studies have highlighted GEM’s involvement in the differentiation of fibroblasts (12, 13), A critical mechanism in the pathogenesis of fibrosis and autoimmune diseases, including SLE. Fibroblast differentiation, particularly into myofibroblasts, is crucial in the development of tissue fibrosis, a common complication in SLE patients, leading to organ damage, especially in the kidneys, skin, and lungs. In the context of SLE, aberrant fibroblast differentiation and excessive extracellular matrix deposition are central to disease progression (14). GEM has been shown to influence the signaling pathways that regulate fibroblast activation and differentiation, potentially contributing to the development of fibrosis and the exacerbation of autoimmunity in SLE (15). The dysregulation of GEM-mediated processes may also be linked to the inflammatory milieu characteristic of SLE, where activated immune cells interact with fibroblasts, leading to tissue damage and chronic inflammation. Despite its potential relevance, research on GEM as a therapeutic target for SLE remains relatively sparse. Although some studies suggest that targeting GEM may mitigate fibroblast differentiation and reduce fibrosis. A considerable gap remains in elucidating the exact molecular mechanisms by which GEM contributes to the pathogenesis of SLE (16). Exploring GEM’s involvement in fibroblast activation and tissue fibrosis may yield crucial insights into the molecular mechanisms of SLE and present novel avenues for developing personalized therapeutic approaches. Targeting GEM in the context of SLE may help to address both the inflammatory and fibrotic aspects of the disease, ultimately improving patient outcomes and advancing the field of precision medicine.

Single-cell technologies have greatly enhanced our comprehension of disease mechanisms, offering in-depth perspectives on cellular heterogeneity and the dynamic processes underlying disease progression (17–19). In SLE, the ability to dissect the differentiation and functional states of various immune and non-immune cell populations is crucial for elucidating the pathogenic mechanisms underlying the disease. SLE is marked by impaired immune responses, such as the activation of autoreactive B and T cells and the development of tissue fibrosis, all of which play a role in the organ damage seen in affected patients. However, the precise cellular mechanisms and the role of distinct cell types in disease progression remain incompletely understood (20–22). Single-cell RNA sequencing (scRNA-seq) has become an essential tool for revealing the cellular landscape of SLE, facilitating the identification of rare and distinct cell populations implicated in inflammatory and fibrotic processes. This technology enables high-resolution gene expression profiling at the single-cell level, essential for studying the disrupted cellular networks and differentiation pathways in SLE. Single-cell technologies enable researchers to monitor cellular differentiation, discover new disease markers, and identify specific molecular targets for therapeutic intervention. In immunology, the advancement of precision therapeutics has become a central focus, utilizing cutting-edge multi-omics technologies to tailor immune treatments. Immunotherapy, a fundamental aspect of precision medicine, manipulates the immune system’s complex dynamics to address a wide range of diseases, from cancer to autoimmune conditions. The integration of multi-omics approaches—encompassing genomics, transcriptomics, proteomics, and metabolomics—offers a comprehensive view of immune responses at the molecular level. Immune signatures derived from these methodologies highlight individual patterns, providing crucial insights into disease susceptibility and therapeutic effectiveness. By merging data from techniques such as single-cell profiling and spatial transcriptomics, we gain a deeper understanding of immune regulation and cellular interactions within disease-specific microenvironments. Together, these efforts aim to tailor therapeutic strategies to each patient’s unique immune landscape, advancing personalized immune modulation and ushering in a new era of precision therapeutics for autoimmune diseases like SLE.

In this study, we apply single-cell sequencing to investigate the role of the GEM gene in SLE. GEM, a GTPase involved in cellular processes such as fibroblast differentiation, has been implicated in fibrotic diseases but remains underexplored in the context of SLE. By leveraging single-cell technologies, we aim to bridge the gap in understanding GEM’s role in the differentiation of fibroblasts and other key cell types involved in SLE pathogenesis. This strategy offers a distinctive opportunity to pinpoint GEM as a potential therapeutic target and to elucidate its molecular mechanisms in SLE, thereby advancing the evolving field of precision medicine for autoimmune disorders.

## 2 Methods

### 2.1 Data source

The scRNA-seq data were retrieved from the GEO database (https://www.ncbi.nlm.nih.gov/geo/) under the GSE accession number GSE179633. As the data utilized in this study were obtained from a publicly available database, ethical approval was not necessary (23).

### 2.2 Processing of single-cell derived data

The gene expression data were processed using R software (v 4.2.0) and analyzed with the Seurat package (v4.1.1). To ensure data accuracy, we employed the DoubletFinder tool (version 2.0.3) to identify and remove potential doublet cells from the dataset (24–26). Low-quality cells were filtered out, and rigorous quality control measures were implemented to ensure the reliability of the single-cell dataset. Cells meeting the following criteria were retained for analysis: 300 < nFeature < 6000 and 500 < nCount < 100,000.

Normalization of all samples was conducted using the “NormalizeData” in the Seurat R package (27–29). After filtering, the top 2,000 most variable genes were identified using the “FindVariableFeatures” function. The generated data were standardized using the ‘ScaleData’ function and subsequently subjected to principal component analysis (PCA) (30, 31) for dimensionality reduction. Harmony R package was used to address batch effects. Subsequently, we utilized Seurat’s “FindClusters” and “FindNeighbors” functions for cell clustering, and “FindAllMarkers” to identify Differentially Expressed Genes (DEGs) (32–35) for each cluster. Finally, we subsequently identified the top 30 principal components for additional analysis and displayed the data with uniform manifold approximation and projection (UMAP) (36–39).

### 2.3 Cell type identification and annotation

We applied the “FindAllMarkers” function to identify differentially expressed genes (DEGs) across various cell clusters (40, 41). Visualizing the expression patterns of these markers using Seurat’s “DotPlot” and “featureplot” tools provided insights into the molecular characteristics of each cluster. Furthermore, our analysis involved re-clustering to delve deeper into the diversity of fibroblasts, focusing on subtype characterization based on unique genetic profiles identified through marker identification techniques.

### 2.4 Enrichment analysis

Gene Ontology (GO) enrichment analysis was conducted using the ClusterProfiler R package (v4.6.0) to identify biological processes and molecular functions associated with differentially expressed genes. GO term significance was assessed with an adjusted P-value threshold of < 0.05. This analysis provided insights into the functional roles of the identified DEGs within the biological context of the study.

### 2.5 Pseudotime analysis

The pseudotemporal trajectory of fibroblast differentiation was reconstructed using the Monocle2 R package (version 2.22.0), a computational framework specifically designed for single-cell trajectory analysis. Differential gene expression analysis was performed to identify statistically significant transcriptional changes (adjusted p-value < 0.05, log2 fold change > 1) across distinct cellular states. The trajectory was constructed using the DDRTree (Discriminative Dimensionality Reduction with Trees) algorithm, which enables dimensionality reduction while preserving the topological relationships between cells. Cellular subtypes were computationally ordered along the pseudotime continuum based on their transcriptional profiles, allowing for the identification of progressive molecular changes during fibroblast differentiation. Genes exhibiting coordinated expression patterns along the pseudotemporal axis were systematically identified through correlation analysis and visualized using a pseudotime heatmap, with hierarchical clustering to reveal co-regulated gene modules.

For lineage inference analysis, the Slingshot R package (version 2.6.0) was implemented to reconstruct the developmental trajectories of fibroblast subpopulations. The “getLineages” function was executed using cluster-based minimum spanning trees (MST) to establish potential differentiation pathways, with cluster identities determined through unsupervised clustering analysis. Subsequently, the “getCurves” function was applied to fit simultaneous principal curves, enabling the quantification of gene expression dynamics across distinct lineages throughout the inferred developmental timeline. The algorithm incorporated cell-level weights to account for uncertainty in cellular state assignment, and pseudotime values were normalized across lineages to facilitate comparative analysis of differentiation kinetics. Both trajectory inference approaches were validated through bootstrapping analysis (n = 1000 iterations) to ensure the robustness of the identified differentiation pathways.

### 2.6 Analysis of cell communication

In this study of cell-cell communication, the CellChat R package (v1.6.1) was employed to analyze intercellular interactions across various cell types. This tool facilitated the construction of regulatory networks based on ligand-receptor interactions, allowing us to infer complex communication patterns. We utilized the “netVisual_diffInteraction” function to visualize the variations in communication strength between cells and employed “identifyCommunicationPatterns” to identify and quantify the distinct intercellular communication patterns. We set a significance threshold of P < 0.05 to evaluate the statistical relevance of these interactions, thereby deepening our understanding of the complex cellular communication networks revealed by our scRNA-seq data.

### 2.7 Gene regulatory landscape

To identify stable cell states and assess transcriptional activity, we employed the pySCENIC package (v0.10.0) in Python (v3.7). Using the AUCell matrix, we evaluated the enrichment of transcription factors (TFs), gaining insights into the functional status of regulatory modules and advancing our understanding of the regulatory landscape across fibroblast subtypes.

### 2.8 Cell culture methodology

CCD-1066 (ATCC^®^ CRL-2096™, RRID: CVCL_1849) and F-2408 (ATCC^®^ CRL-12047™, RRID: CVCL_1060) cell lines were cultured in Dulbecco’s Modified Eagle Medium (DMEM) (Gibco, Thermo Fisher Scientific, Cat. No. 11965092) supplemented with 10% fetal bovine serum (FBS) (Gibco, Thermo Fisher Scientific, Cat. No. 16000044) and 1% penicillin-streptomycin (Gibco, Thermo Fisher Scientific, Cat. No. 15140122). Cells were maintained at 37°C in a 5% CO2 incubator and the medium was refreshed every 2-3 days. When cultures reached 80-90% confluence, cells were trypsinized using 0.25% trypsin-EDTA (Gibco, Thermo Fisher Scientific, Cat. No. 25200056) and subcultured at appropriate densities for subsequent experiments.

### 2.9 Gene knockdown by transfection of CCD-1066 and F-2408 cells

Gene knockdown of GEM in CCD-1066 (ATCC^®^ CRL-2096™, RRID: CVCL_1849) and F-2408 (ATCC^®^ CRL-12047™, RRID: CVCL_1060) cells was performed using GEM-targeting siRNA (siGEM) and non-targeting control siRNA (siControl) obtained from GenePharma. Cells were seeded in 6-well plates at 70-80% confluence and transfected with 50 nM siRNA using Lipofectamine 3000 reagent (Invitrogen, Thermo Fisher Scientific, Cat. No. L3000008) following the manufacturer’s instructions. After 6 hours, the medium was replaced with complete growth medium (DMEM, 10% FBS, 1% penicillin-streptomycin). Cells were harvested 48-72 hours post-transfection for RNA and protein extraction. GEM knockdown efficiency was assessed by qRT-PCR using PrimeScript RT Reagent Kit (Takara, Cat. No. RR047A) and SYBR Green Master Mix (Applied Biosystems, Cat. No. 4367659), and confirmed by Western blotting with anti-GEM antibody (Santa Cruz Biotechnology, Cat. No. sc-365839, RRID: AB_10835764).

### 2.10 RT-qPCR analysis of GEM gene expression in CCD-1066 and F-2408 cells

Total RNA was extracted from CCD-1066 (ATCC^®^ CRL-2096™, RRID: CVCL_1849) and F-2408 (ATCC^®^ CRL-12047™, RRID: CVCL_1060) cells using the RNeasy Mini Kit (Qiagen, Cat. No. 74104). RNA quality and quantity were assessed with a NanoDrop spectrophotometer (Thermo Fisher Scientific). Reverse transcription was performed using the PrimeScript RT Reagent Kit (Takara, Cat. No. RR047A) following the manufacturer’s instructions. cDNA was then analyzed by quantitative PCR using SYBR Green Master Mix (Applied Biosystems, Cat. No. 4367659) on an Applied Biosystems 7500 real-time PCR system (Applied Biosystems, RRID: SCR_015135). The PCR conditions included an initial denaturation at 95°C for 10 minutes, followed by 40 cycles of 95°C for 15 seconds and 60°C for 1 minute. Gene expression of GEM (forward primer: 5’-GGAAGAGCTGGAAAGGCTGA-3’, reverse primer: 5’-TGAGCCAGGGCGATAGAACT-3’) was normalized to GAPDH (forward primer: 5’-GAAGGTGAAGGTCGGAGTC-3’, reverse primer: 5’-GAAGATGGTGATGGGATTTC-3’) as an internal control. Relative expression levels were calculated using the ΔΔCt method, and statistical significance was assessed using appropriate tests, with p-values < 0.05 considered significant.

### 2.11 CCK-8 assay for cell viability in CCD-1066 and F-2408 cells

Cells were seeded in 96-well plates at a density of 1 × 10³ cells per well. After incubation at 37°C for 2 hours, CCK-8 reagent (Vazyme, A311-01) was added to the wells. Cell viability was measured by assessing absorbance at 450 nm using an enzyme-linked spectrophotometer (Thermo, A33978) at 0, 24, 48, 72, and 96 hours.

### 2.12 Colony formation assay in CCD-1066 and F-2408 cells

CCD-1066 and F-2408 cells were seeded in six-well plates at a density of 500–1,000 cells per well and cultured for 7–10 days in complete growth medium (DMEM with 10% FBS and 1% penicillin-streptomycin). Colonies were fixed with 4% paraformaldehyde for 15 minutes and stained with 0.5% crystal violet for 30 minutes. Excess stain was removed with PBS, and colonies with ≥50 cells were counted using a light microscope. Colony numbers were compared between experimental and control groups, with statistical significance determined at p < 0.05.

### 2.13 Wound healing assay in CCD-1066 and F-2408 cells

CCD-1066 and F-2408 cells were seeded in six-well plates and cultured to confluence in complete growth medium (DMEM with 10% FBS and 1% penicillin-streptomycin). A scratch was created using a sterile 200 µL pipette tip, and debris was removed by washing with PBS. Cells were then incubated in serum-free medium for 24 hours. Wound closure was imaged at 0 and 24 hours using a light microscope, and the wound area was quantified with ImageJ software. Migration rate was calculated as the percentage of wound closure.

### 2.14 Transwell migration assay in CCD-1066 and F-2408 cells

CCD-1066 and F-2408 cells (1×10^5^) were suspended in serum-free DMEM and seeded into the upper chamber of a 24-well Transwell plate with an 8 µm pore size membrane. The lower chamber contained complete DMEM with 10% FBS as a chemoattractant. After 24 hours of incubation at 37°C in 5% CO2, cells that migrated to the lower surface were fixed with methanol, stained with crystal violet, and counted under a light microscope. The number of migrated cells was averaged from five random fields per well.

### 2.15 Flow cytometry analysis of apoptosis in CCD-1066 and F-2408 cells

Apoptosis in CCD-1066 and F-2408 cells was evaluated using the Annexin V-FITC Apoptosis Detection Kit. Cells (1×10^6^) were seeded in 6-well plates and treated accordingly. After 24 hours, cells were harvested, washed with PBS, and resuspended in 1× binding buffer. The suspension was stained with 5 μL Annexin V-FITC and 5 μL propidium iodide (PI) for 15 minutes at room temperature in the dark. Apoptosis was analyzed using a BD FACSCanto II flow cytometer, with data acquired via BD FACSDiva software. Apoptotic rates were calculated as the percentage of Annexin V-positive cells.

### 2.16 Statistical analysis

Statistical analyses were conducted using GraphPad Prism 9.0. Data normality was assessed with the Shapiro-Wilk test. Comparisons between two groups were made using an unpaired Student’s t-test, while multiple comparisons were analyzed by one-way or two-way ANOVA, followed by Tukey’s *post-hoc* test. Results are expressed as mean ± standard deviation (SD). A p-value < 0.05 was considered statistically significant, with all analyses performed at a 95% confidence level.

## 3 Results

### 3.1 Visualization of fibroblast subtypes

**Figure 1:**
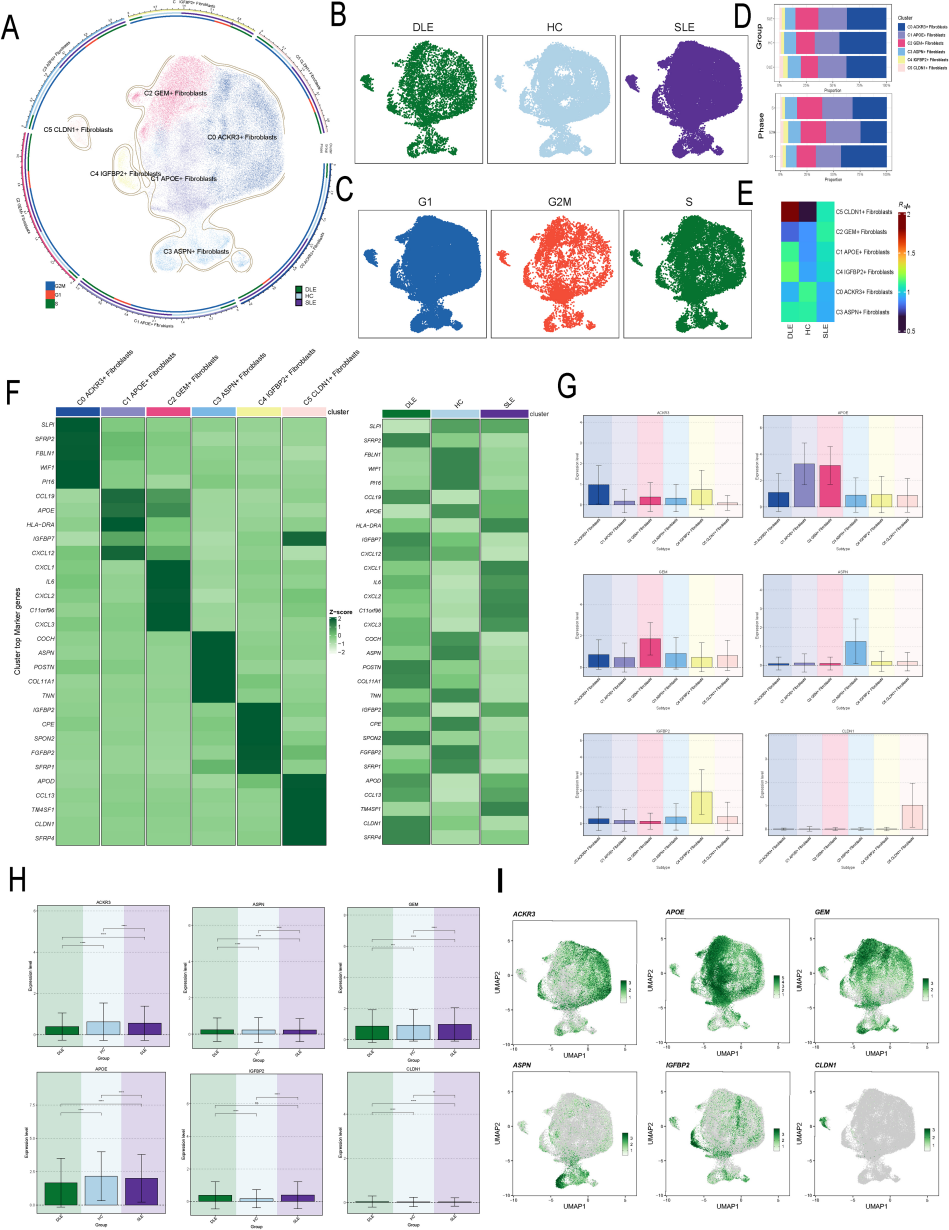
Visualization and Characterization of Fibroblast Subtypes in Disease Contexts. **(A)** The UMAP plot illustrated the distribution of six fibroblast subtypes. The outer circle represents the total cell count for each subtype, while the middle and inner circles show the proportions of cell cycle and group assignments, respectively. **(B)** The UMAP plot illustrated the distribution of disease groups in fibroblasts. **(C)** The UMAP plot illustrated the distribution of cell cycles in fibroblasts. **(D)** The bar plots showed the proportion of fibroblast subtypes in different cell cycles and disease groups. **(E)** The Ro/e score was used to evaluate the grouping preferences of each fibroblast subtype. **(F)** The heatmap displayed the top 5 differentially expressed genes in each fibroblast subtype. **(G, H)** The bar plot displayed the distribution of the C2 subtype marker gene GEM across different fibroblast subtypes and disease groups. The symbols represent the following significance levels: ** : P < 0.01, *** : P < 0.001, **** : P < 0.0001. ns : Not significant (P≥0.05). **(I)** The UMAP plot showed the distribution of characteristic marker genes for the six fibroblast subtypes.

Fibroblasts were grouped into 6 clusters, and were ultimately annotated as C0 ACKR3+ Fibroblasts, C1 APOE+ Fibroblasts, C2 GEM+ Fibroblasts, C3 ASPN+ Fibroblasts,C4 IGFBP2+ Fibroblasts and C5CLDN1+ Fibroblasts (**Figure 1A**). Additionally, we visualized the distribution of fibroblasts based on disease groups and cell cycle phases (**Figures 1B, C**). And analysis of the proportions of different fibroblast subtypes across disease groups revealed that, compared to HC and DLE groups, C2 had a higher proportion in SLE (**Figure 1D**). Additionally, the Ro/e score indicated that C2 are more likely to be associated with SLE, suggesting a correlation between C2 and the pathogenesis of SLE (**Figure 1E**). We further analyzed the top 5 marker genes for each subtype and their expression across disease groups, revealing that C2 markers are significantly upregulated in SLE. (**Figure 1F**). Subsequently, we analyzed the expression of subtype-specific marker gene across subtypes and disease groups (**Figures 1G, H**) and mapped their distribution within the fibroblasts (**Figure 1I**). Notably, we observed a significant overlap between the distribution of GEM, the subtype-specific marker genes of C2, and SLE. The elevated expression of C2 marker genes in SLE might provide additional evidence supporting the strong link between C2 and SLE pathology.

### 3.2 Functional analysis of fibroblast subtypes

**Figure 2:**
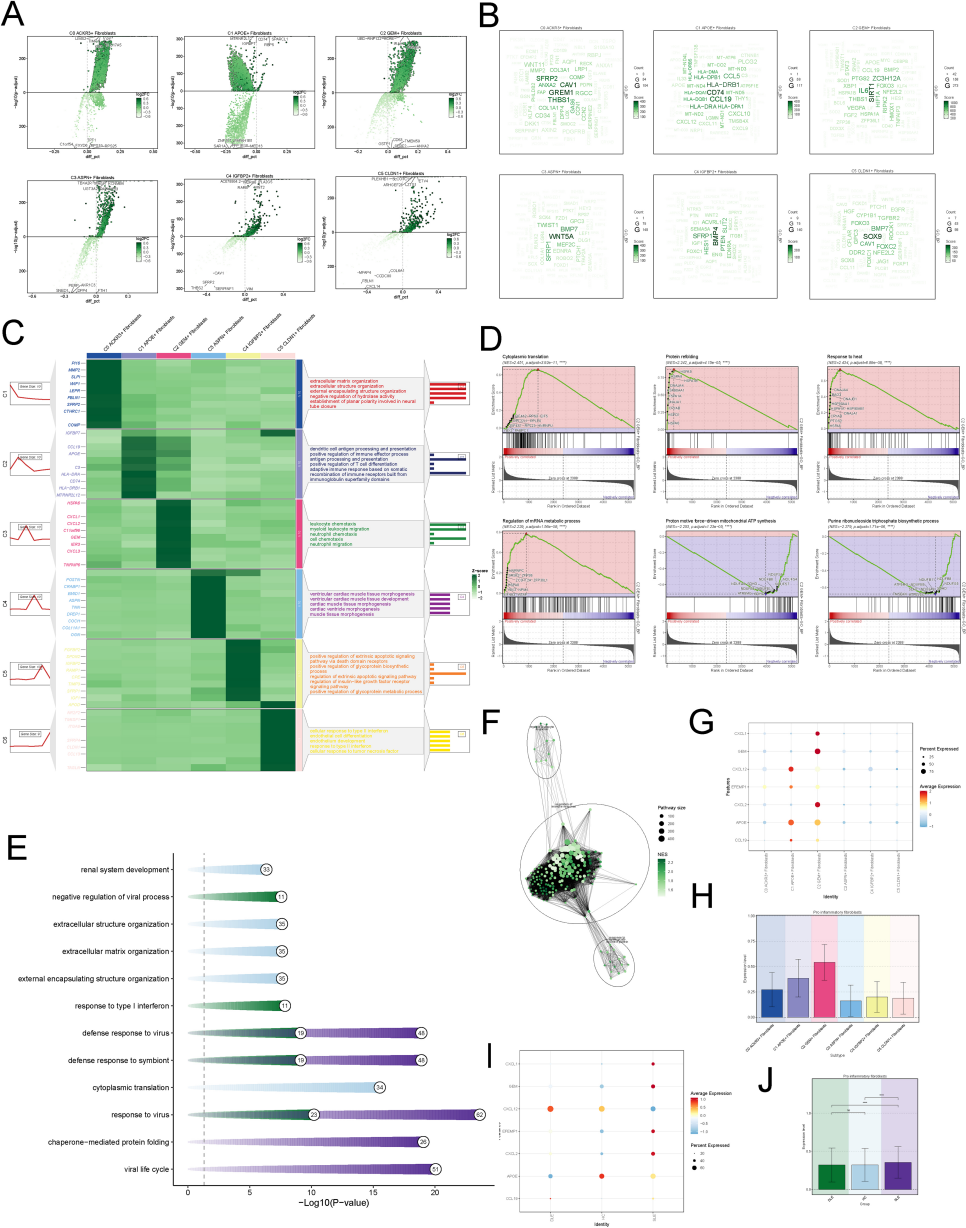
Functional Characterization of Fibroblast Subtypes **(A)** Volcano maps of top 5 up-regulated and top 5 down-regulated genes in fibroblasts subtypes. **(B)** The word cloud maps exhibited gene enrichment of fobroblasts subsets. **(C)** The heatmap exhibited the genes enriched by various subsets of fibroblast and the GOBP pathway. **(D)** GSEA of C2 subtype to identify key biological pathways. **(E)** The bar plot showed the enriched pathways across different disease groups. **(F)** The network plot exhibited the enriched pathways in SLE group. **(G)** The bubble plot displayed the expression of pro-inflammatory genes across different cell subtypes. The size of the bubbles represented the percentage of gene expression, while the color indicateed normalized data. **(H)** The bar plots displayed the pro-inflammatory scores across different fibroblast subtypes. **(I)** The bubble plot displayed the expression of pro-inflammatory genes across different disease groups. **(J)** The bar plots displayed the pro-inflammatory scores across different disease groups. The symbols represent the following significance levels: **** : P < 0.0001. ns : Not significant (P≥0.05).

To explore the functional characteristics of different fibroblasts, we analyzed the differential genes across various fibroblast subtypes (**Figure 2A**).We found that GREM1, CAV1, and THBS1 were highly correlated with C0; HLA-DRB1, CD74, and CCL19 were strongly associated with C1; SIRT1, IL16, and ZC3H12A were closely linked to C2; WNT5A, BMP4, and SFRP1 were highly correlated with C3; BMP4, SFRP1, and PTEN were associated with C4; and SOX9, BMP7, and FOXC2 were highly correlated with C5 (**Figure 2B**). The GO enrichment analysis of fibroblast subtypes showed that C0 is primarily associated with the extracellular matrix organization, C1 with antigen processing and presentation, C2 with leukocyte chemotaxis and migration, C3 with muscle tissue morphogenesis, C4 with apoptotic signaling pathway, and C5 with type II interferon response and endothelial cell differentiation (**Figure 2C**). We subsequently performed gene set enrichment analysis (GSEA) on C2, revealing upregulation of biological processes such as cytoplasmic translation and protein refolding. These findings may reflect heightened cellular activity during disease progression (**Figure 2D**). Further enrichment analysis based on disease groups revealed that fibroblasts in the HC group were predominantly enriched in extracellular structure organization, whereas fibroblasts in the disease groups were primarily enriched in defense response (**Figure 2E**). Additionally, the SLE group exhibited enrichment in pathways related to immune response, further highlighting the involvement of immunological mechanisms in disease pathology(**Figure 2F**).

We specifically visualized the expression levels of pro-inflammatory genes in each fibroblast subtype and found that C2 expressed higher levels of pro-inflammatory genes (**Figure 2G**), with the highest scores in the pro-inflammatory scoring (**Figure 2H**). Additionally, compared to the HC and DLE groups, fibroblasts in the SLE group also exhibited elevated pro-inflammatory levels (**Figures 2I, J**).

### 3.3 Fibroblast subtype development and differentiation

To further understand the origins and development of fibroblast subtypes, we first used Monocle to determine the differentiation trajectory of fibroblast subpotypes.

**Figure 3:**
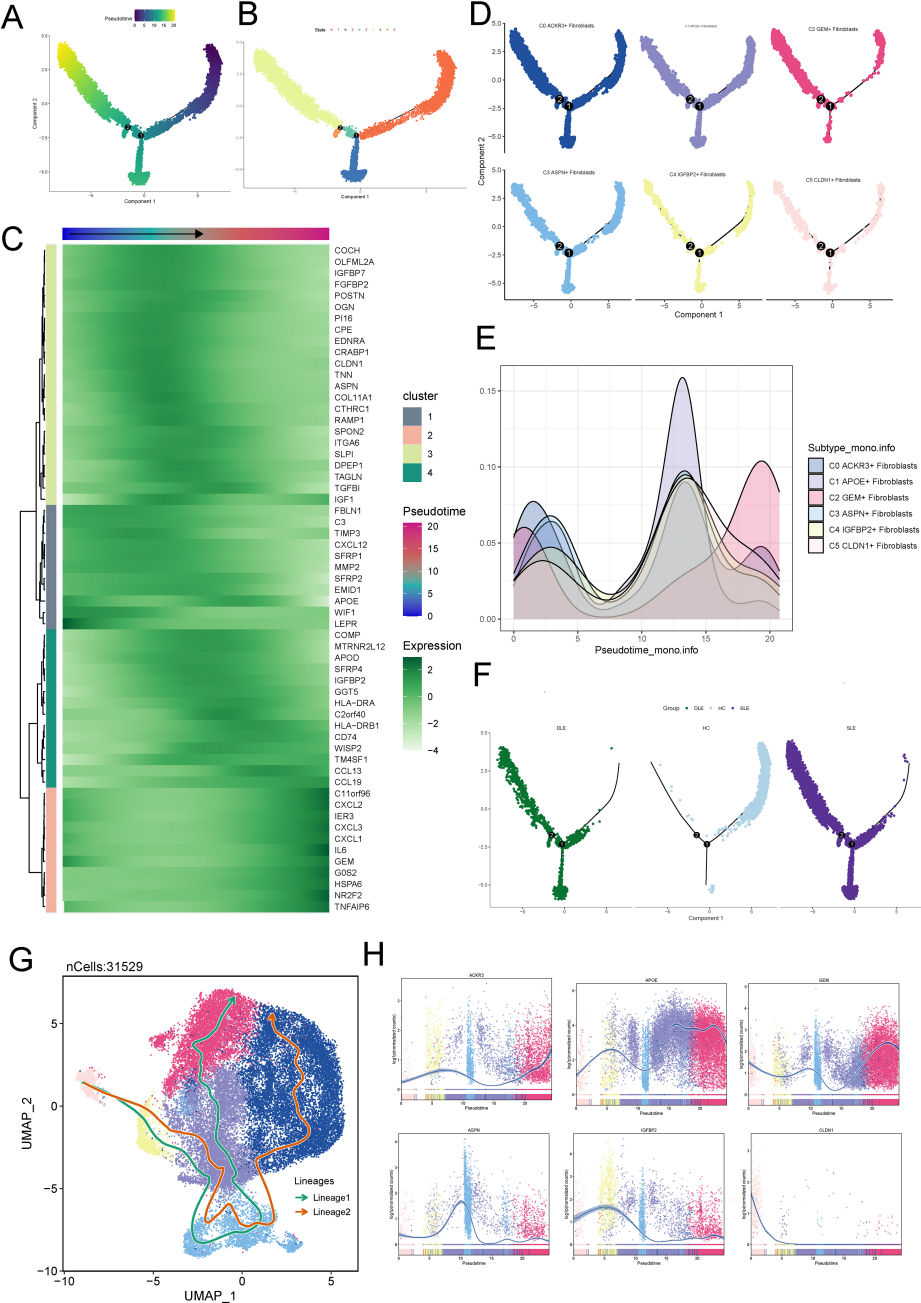
Fibroblast Subtype Development and Differentiation. **(A)** General pseudotime differentiation trajectory of fibroblast subtypes analyzed using Monocle. **(B)** Pseudotime trajectory showing differentiation from the starting point (upper right corner) to differentiation point 1 (state 1). From state 1, two branches emerged: one leading to state 2 (lower right corner) and the other splitting into state 3 (upper left corner), which further branches into state 4 (upper left) and state 5 (lower left). **(C)** Expression levels of differentiation-associated genes (CD44, TWIST1, NES, NOTCH1, HIF1A, EPAS1) during the fibroblast differentiation process, indicating increasing expression as differentiation progresses. **(D, E)** Visualization of fibroblast subtypes along the pseudotime trajectory, with the C2 subtype predominantly localized in the later stages of differentiation. **(F)** Comparison of fibroblast subtype distribution in SLE and DLE skin lesions versus HC, showing a higher concentration of fibroblasts at the later stages of pseudotime in disease samples, correlating with disease progression. **(G)** UMAP plots displaying two distinct fibroblast lineage trajectories inferred using Slingshot: Lineage 1 (C5-C4-C3-C1-C2) and Lineage 2 (C5-C4-C1-C3-C0). Lineage 2, with C2 at the terminal stage, is suggested to represent the differentiation pathway associated with SLE. **(H)** Expression of fibroblast subtype-specific marker genes along the pseudotime trajectory.

In **Figure 3A** we showed the general pseudotime differentiation trajectory. The overall pseudotime trajectory (**Figure 3B**), with the upper right corner as the starting point, differentiated to the lower left to differentiation point 1, which was called state 1. From differentiation point 1, it was divided into two branches, and one branch differentiated to the lower right corner, which was called state 2; the other branch differentiated to upper left corner to differentiation point 2, which was called state 3; a branch extending from differentiation point 2 to the upper left was called state 4; and a branch extending from the lower left was called state 5. Besides, we observed that the expression of ACXCL2, IER3, CXCL3, IL6, GOS2, HSPA6, and NR2F2 increased during the differentiation process (**Figure 3C**).We further visualized the fibroblast subtypes along the pseudotime trajectory and observed that, compared to other subtypes, the C2 subtype was predominantly localized at the later stages of differentiation (**Figures 3D, E**). Finally, we observed that, compared to HC, fibroblasts in the skin lesions of SLE and DLE patients were more concentrated at the later stages of the pseudotime trajectory (**Figure 3F**), which is consistent with the progression of the disease.

Subsequently, Slingshot was employed to infer the differentiation pathways of fibroblast subtypes. We utilized UMAP plots to separate two cell lineage trajectories (**Figure 3G**). Including: lineage 1:C5-C4-C3-C1-C2; lineage 2:C5-C4-C1-C3-C0. We supposed that lineage 2 represented the differentiation lineage related to SLE, as C2 was at the end of the lineage, and it may represent the course of the disease. Then, we analyzed the expression of different marker genes along the overall pseudotime trajectory. It was observed that the marker gene IGFBP2 for C4 and the marker gene APOE for C1 were primarily expressed in the early stages of the differentiation trajectory, while the marker gene GEM for C2 was predominantly expressed in the later stages of differentiation (**Figure 3H**).

### 3.4 Cell stemness analysis of fibroblast subtypes

**Figure 4:**
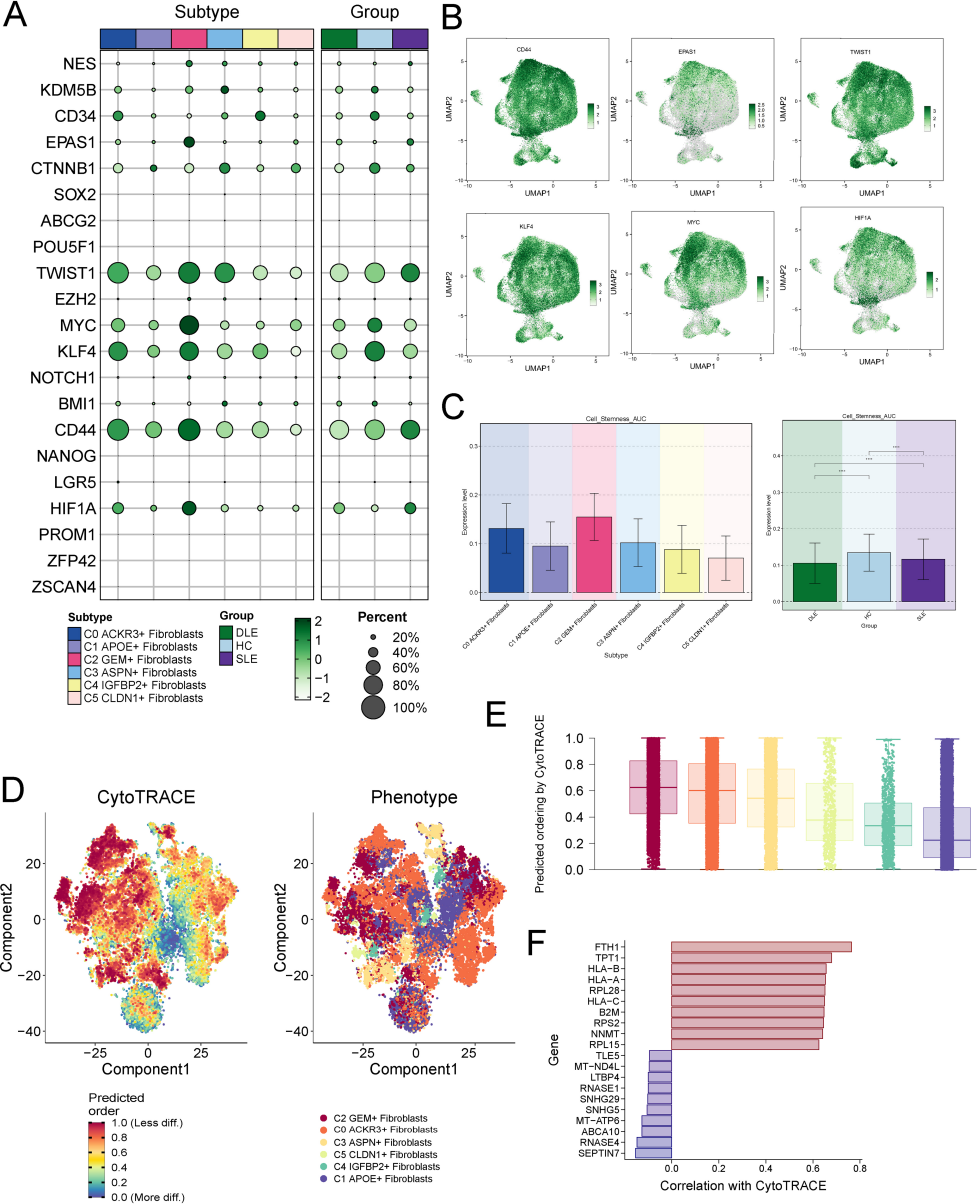
Cell Stemness Analysis of Fibroblast Subtypes. **(A)** Expression of stemness-associated genes across different fibroblast subtypes, with a focus on C2 showing elevated levels of stemness markers. **(B)** Expression levels of key stemness genes (TWIST, MYC, HIF1A, EPAS1, KLF4, CD44) in fibroblast subtypes, with C2 exhibiting significantly higher expression compared to other subtypes. **(C)** AUC scores for stemness, confirming that C2 exhibits the highest activity of stemness-related genes, indicating greater differentiation potential. The symbols represent the following significance levels: ****: P < 0.0001. **(D, E)** CytoTRACE analysis showing that C2 fibroblasts have the highest differentiation potential, consistent with their stemness AUC scores. **(F)** Gene mining approach in CytoTRACE identifying members of the HLA gene family (HLA-B, HLA-A, HLA-C) as strongly correlated with stemness, highlighting the immune-functional characteristics of C2 fibroblasts.

To investigate the development and differentiation of fibroblasts, we first analyzed the expression of stemness-associated genes across the fibroblast subtypes (**Figure 4A**). Notably, compared to other subtypes, the stemness genes CD44, EPAS1, TWIST1, KLF4, MYC and HIF1 exhibited higher expression levels in C2 (**Figure 4B**). Similarly, the AUC score for stemness further confirmed that C2 had the highest scores, indicating higher activity of activity of stemness-related genes and greater differentiation potential in C2 (**Figure 4C**).

We then employed CytoTRACE to further assess the differentiation potential of each fibroblast subtype. Consistent with the stemness AUC scores, C2 exhibited the highest CytoTRACE scores, suggesting enhanced stemness and differentiation capacity (**Figures 4D, E**). Subsequently, we applied the gene mining approach in CytoTRACE to identify genes associated with the differentiation levels of fibroblasts. This analysis revealed that members of the HLA gene family, including HLA-B, HLA-A, and HLA-C, were significantly correlated with stemness (**Figure 4F**). As key players in immune recognition, the HLA genes underscore the immune-functional characteristics of C2 subtype.

### 3.5 CellChat analysis among all cells and key signaling crosstalk between the C2 subtype and other cells

**Figure 5:**
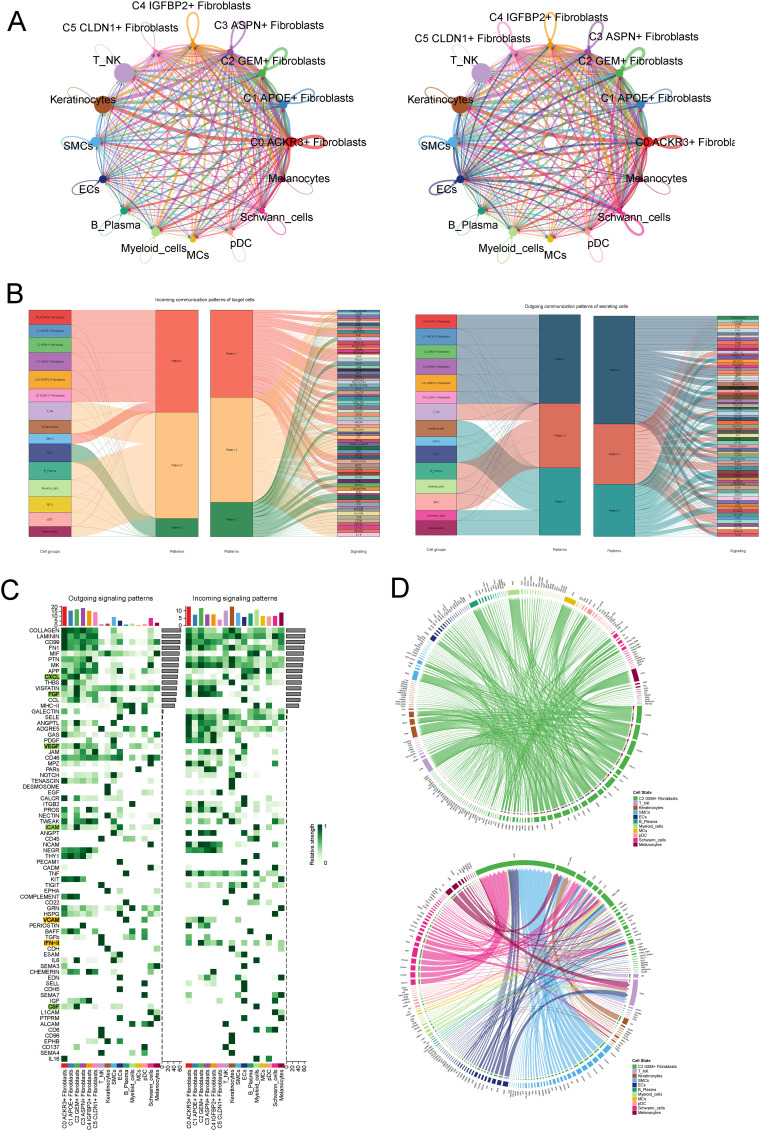
CellChat Analysis of Intercellular Communication and Crosstalk in the C2 Subtype. **(A)** Visualization of the number and strength of intercellular interactions across all cell populations, including fibroblasts. **(B)** Hierarchical clustering analysis revealing three distinct outgoing and incoming communication patterns. Fibroblasts were categorized under outgoing pattern 1, characterized by the secretion of ligands involved in key signaling pathways, such as COLLAGEN, LAMININ, and CD99, and incoming pattern 1, modulated by signaling molecules like CD99, PTN, and MK. **(C)** Expression analysis of outgoing and incoming signaling pathways across various cell types, highlighting upregulation of CXCL, FGF, VEGF, and ICAM pathways in the outgoing signaling of C2, and VCAM and IFN II pathways in the incoming signaling. **(D)** Circular plots visualizing the communication patterns of the C2 fibroblast subtype with other cell types, illustrating both outgoing and incoming signaling interactions.

We utilized cellchat to investigate intercellular communication between all kinds of cells including fibroblasts and other cell types. Initially, we visualized the number and strength of interactions among different cell populations (**Figures 5A, B**). Subsequently, we analyzed the outgoing and incoming communication patterns of each cell subtype, corresponding to ligand and receptor expression profiles, respectively. Through hierarchical clustering analysis, we identified three distinct outgoing patterns and three incoming communication patterns. Fibroblasts were predominantly categorized under outgoing pattern 1, characterized by the secretion of ligands associated with key signaling pathways, including COLLAGEN, LAMININ and CD99. Similarly, all fibroblast subtypes were classified under incoming pattern 1, primarily modulated by signaling pathways such as CD99, PTN, and MK (**Figure 5C**). Furthermore, we analyzed the expression levels of outgoing and incoming signaling pathways across different cell types (**Figure 5D**). Notably, the CXCL, FGF, VEGF, and ICAM pathways were upregulated in the outgoing signaling of C2 subtype, while VCAM and IFN II pathways were upregulated in the incoming signaling of C2. Additionally, we visualized the communication patterns of the C2 subtype with other cell types in both outgoing and incoming signaling patterns using circular plots (**Figure 5E**).

**Figure 6:**
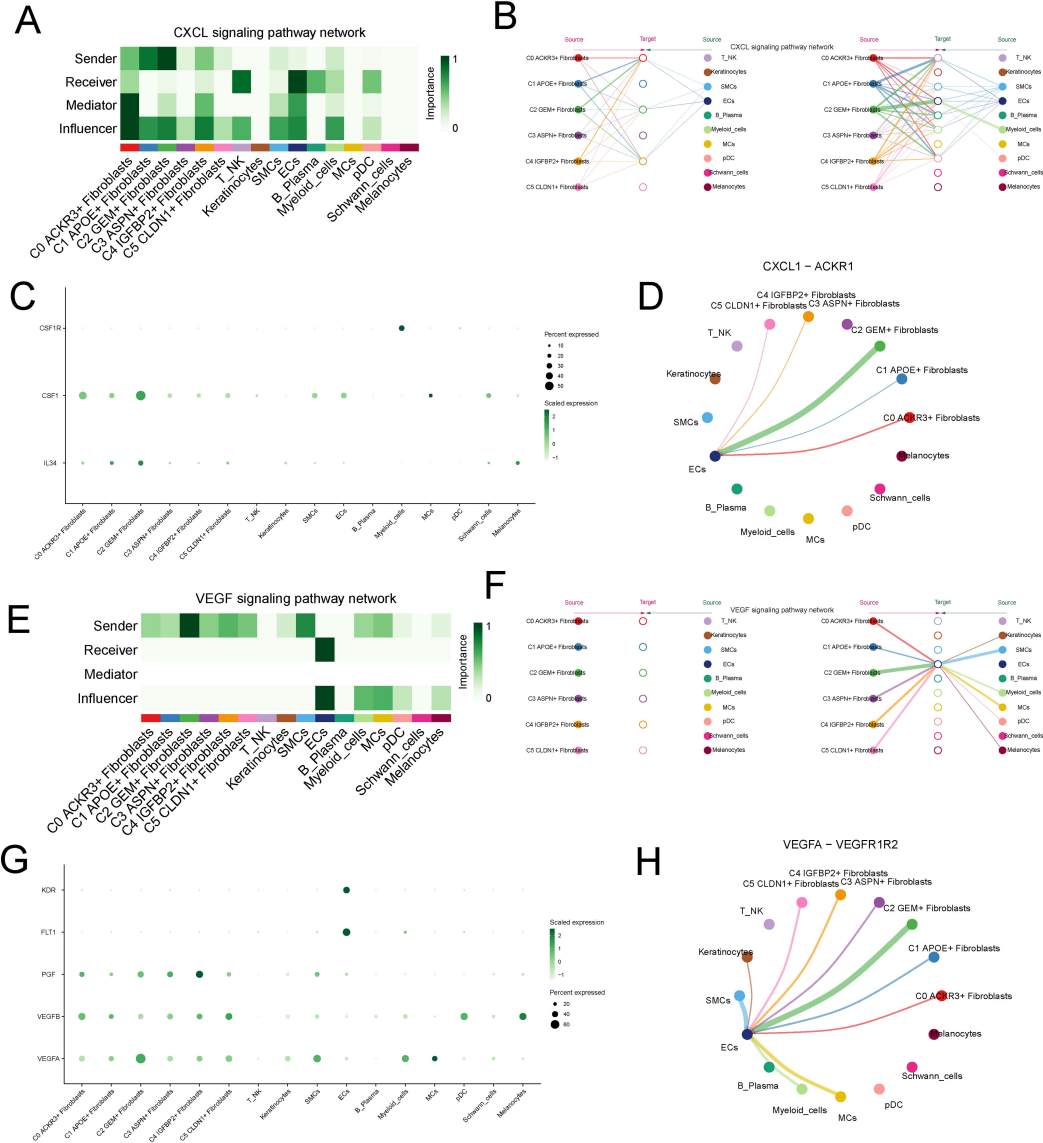
Analysis of Upregulated CXCL and VEGF Signaling Pathways in the C2 Subtype. **(A, B)** Visualization of the CXCL signaling pathway, predominantly secreted by the C2 fibroblast subtype and acting on endothelial cells in a paracrine manner. **(C, D)** Interaction between the CXCL1 ligand, secreted by C2 fibroblasts, and the ACKR receptor expressed on endothelial cells. **(E, F)** Visualization of the VEGF signaling pathway, secreted by the C2 subtype and acting on endothelial cells in a paracrine manner. **(G, H)** Interaction between the VEGFA1 ligand, secreted by C2, and its receptors VEGFR1 and VEGFR2 on endothelial cells.

In the outgoing signaling pathways, we selected the significantly upregulated CXCL and VEGF pathways in the C2 subtype for further analysis. The CXCL signaling pathway was predominantly secreted by the C2 subtype and primarily acted on endothelial cell in a paracrine mode (**Figures 6A, B**). In the CXCL signaling pathway, the ligand CXCL1, secreted by C2, interacted with the receptor ACKR expressed on endothelial cell (**Figures 6C, D**).The VEGF signaling pathway is primarily secreted by the C2 subtype and acted on endothelial cell in a paracrine mode (**Figures 6E, F**). In the VEGF pathway, the ligand VEGFA1, secreted by C2, interacted with the receptors VEGFR1 and VEGFR2 on endothelial cells (**Figures 6G, H**).

**Figure 7:**
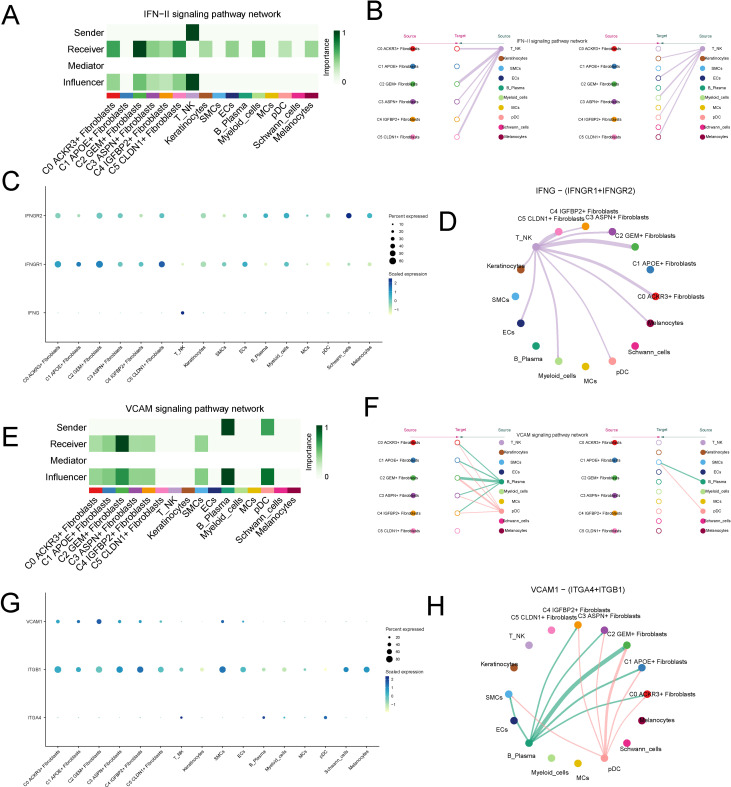
Analysis of Upregulated IFN-II and VCAM Signaling Pathways in the C2 Subtype. **(A, B)** Visualization of the IFN-II signaling pathway, predominantly secreted by NK cells and acting on the C2 fibroblast subtype in a paracrine manner. **(C, D)** Interaction between the IFNG ligand, secreted by NK cells, and the IFNGR1 receptor expressed on the C2 fibroblast subtype. **(E, F)** Visualization of the VCAM signaling pathway, secreted by plasma cells and acting on the C2 subtype in a paracrine manner. **(G, H)** Interaction between the VCAM1 ligand, secreted by plasma cells, and the ITGB1 receptor on the C2 fibroblast subtype.

In the incoming signaling pathways, the significantly upregulated IFN-II and VCAM pathways in C2 were selected for further analysis. The IFN-II signaling pathway was primarily secreted by NK cell and mainly acted on the C2 subtype in a paracrine mode (**Figures 7A, B**). In the IFN-II signaling pathway, the ligand IFNG, secreted by NK cell, interacted with the receptor IFNGR1 expressed on the C2 subtype (**Figures 7C, D**). The VCAM pathway was primarily secreted by plasma cell and acted on the C2 subtype in a paracrine mode (**Figures 7E, F**). In the VCAM pathway, the ligand VCAM1, secreted by plasma cells, interacted with the receptor ITGB1 on the C2 subtype (**Figures 7G, H**).

### 3.6 Potential regulatory mechanism of C2 subtype

**Figure 8:**
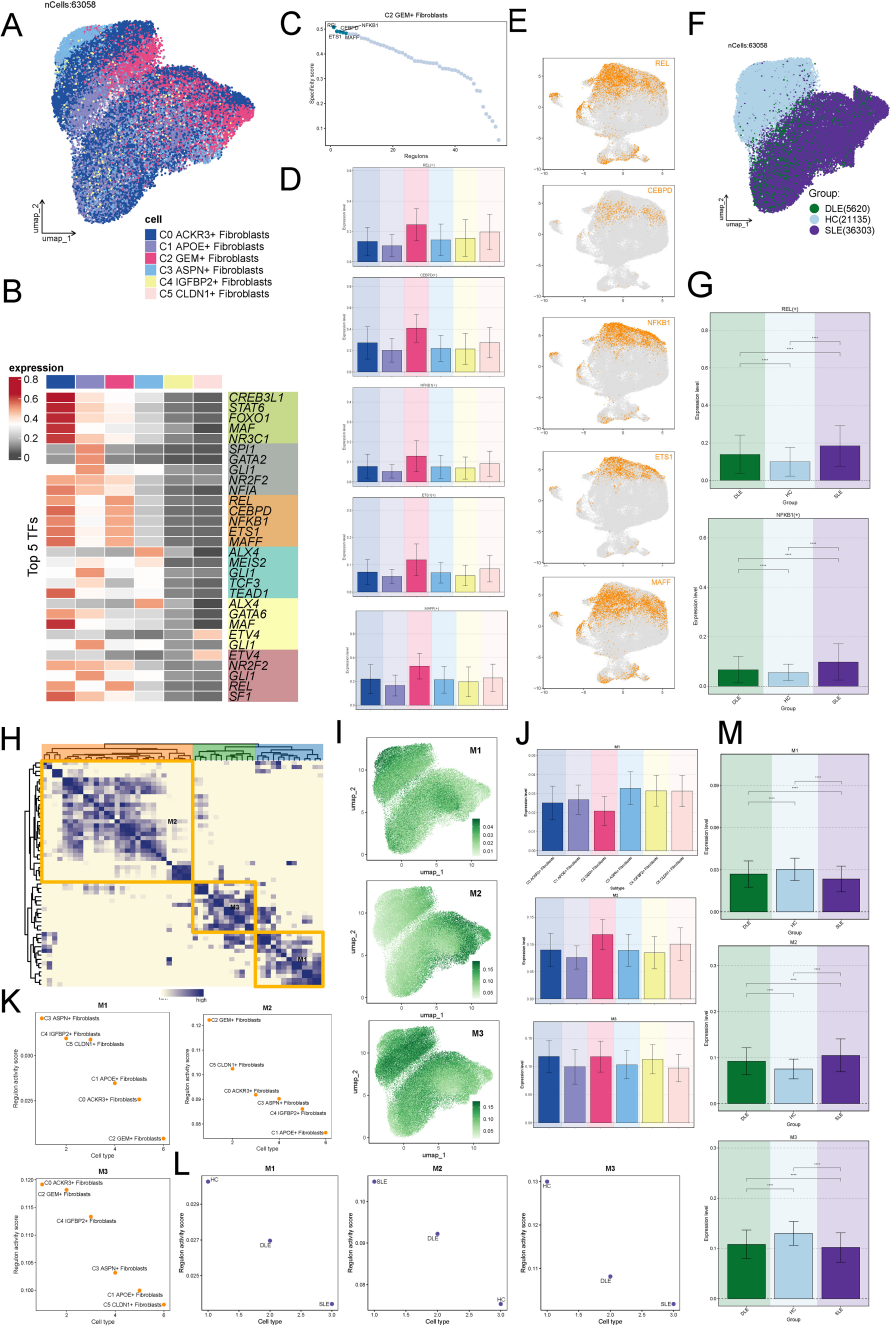
Potential Regulatory Mechanisms of the C2 Subtype. **(A)** UMAP clustering revealing the distribution of transcription factors across fibroblast subtypes. **(B)** Identification of the top 5 ranked transcription factors for each fibroblast subtype. **(C)** Top 5 regulons for the C2 fibroblast subtype based on the RSS analysis: REL, CEBP, NFKB1, ETS1, and MAFF. **(D, E)** Visualization of the top regulons (REL, CEBP, NFKB1, ETS1, MAFF) across the fibroblast subtypes. **(F)** Distribution of transcription factors across the HC, DLE, and SLE groups, highlighting significant overlap between SLE and C2. **(G)** Higher expression levels of REL and NFKB1 regulons in the SLE group, confirming the similarity with the C2 subtype. The symbols represent the following significance levels: ****: P < 0.0001. **(H)** Clustering of cells into three modules (M1-M3) based on the regulons identified by pySCENIC. **(I)** Comparison of RSS values across fibroblast subtypes in the three modules, with higher RSS observed for C2 in module M2. **(J, K)** Visualization of the regulon distribution showing greater overlap between M2 and C2 fibroblast subtype. **(L)** Higher RSS in M2 for SLE compared to HC and DLE, supporting the hypothesis of shared transcriptional regulatory characteristics. **(M)** Expression levels of different modules across the groups, with module M2 showing higher expression in SLE. The symbols represent the following significance levels: ****: P < 0.0001.

We applied pySCENIC to derive the RAS matrix. First, we performed unsupervised clustering using the UMAP algorithm, which revealed the distribution of transcription factors across the fibroblast subtypes (**Figure 8A**). We then identified the top 5 ranked transcription factors for each fibroblast subtype (**Figure 8B**). Focusing on the C2 subtype, we identified the top 5 regulons based on RSS: REL, CEBP, NFKB1, ETS1, and MAFF (**Figure 8C**). We then visualized these top regulons in (**Figures 8D, E**). Subsequently, we visualized the distribution of transcription factors across the HC, DLE, and SLE groups (**Figure 8F**). It was evident that there was significant overlap between the transcription factors of SLE and C2, suggesting that fibroblasts in SLE may share similar transcriptional regulatory characteristics with C2. Further analysis revealed that the regulons REL and NFKB1 exhibited the highest expression in SLE (**Figure 8G**), which confirming this hypothesis.

Additionally, based on the CSI of the regulons identified by pySCENIC, we clustered the cells into three modules (M1-M3) (**Figure 8H**). By comparing the RSS of fibroblasts subtypes across the modules, we observed that the RSS of C2 was higher in M2 (**Figure 8I**). Additionally, we visualized the distribution of regulons and observed a greater overlap between M2 and C2 (**Figures 8J, K**). We further observed that the RSS of M2 in SLE was also significantly higher compared to HC and DLE (**Figure 8L**), supporting the hypothesis that C2 and SLE might share similar transcriptional regulatory processes (**Figure 8L**). Finally, by visualizing the expression levels of different modules across the groups, we found that, consistent with previous results, M2 showed higher expression in SLE (**Figure 8M**).

### 3.7 GEM gene knockdown significantly inhibits abnormal activity of fibroblasts

**Figure 9:**
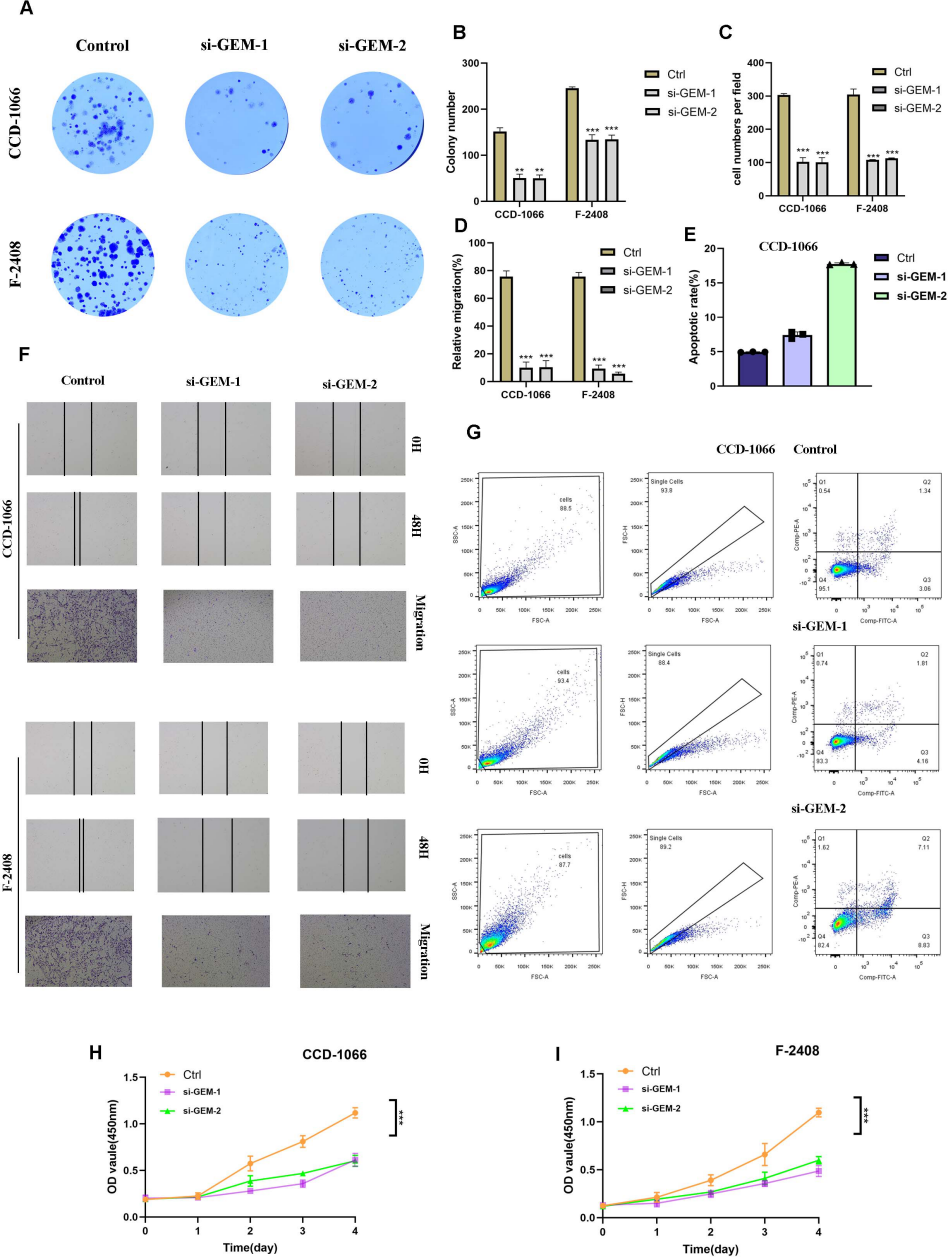
Effect of GEM Gene Knockdown on Fibroblast Activity. **(A, B)** Colony formation assays demonstrating that GEM knockdown The symbols represent the following significance levels: significantly reduced colony size in both CCD-1066 and F-2408 fibroblasts, indicating inhibited fibroblast proliferation. **(B–D, F)** Quantification of colony formation assays, transwell migration assays and Scratch wound healing assays showing that GEM knockdown significantly suppressed fibroblast proliferation and migratory capacity. The symbols represent the following significance levels: ** : P< 0.01, *** : P<0.001. **(E, G)** Flow cytometry analysis revealing that GEM knockdown significantly enhanced apoptosis in both CCD-1066 and F-2408 fibroblasts. **(H, I)** CCK-8 assay confirming that GEM knockdown reduced cell viability in both CCD-1066 and F-2408 fibroblasts.

To investigate the effect of GEM gene knockdown on fibroblasts, we transfected CCD-1066 and F-2408 fibroblasts with siRNA targeting GEM, the transfection efficiency was verified by RT-qPCR (**Supplementary Figure 1**) and performed colony formation assays. The results indicated that GEM knockdown significantly reduced the colony size in both CCD-1066 and F-2408 fibroblasts, suggesting that GEM suppression inhibited fibroblast proliferation (**Figures 9A, B**).

To further validate these findings, we assessed the cell viability of CCD-1066 and F-2408 fibroblasts using the CCK-8 assay, which confirmed that GEM knockdown reduced the viability of both cell lines (**Figures 9H, I**).

Next, we examined the effect of GEM on fibroblast migration using scratch wound healing and transwell assays. The data revealed that silencing GEM notably impaired the migratory capacity of CCD-1066 and F-2408 fibroblasts (**Figures 9C, D, F**). These results indicate that GEM knockdown inhibits both the proliferation and migration of fibroblasts.

Since abnormal fibroblast proliferation is a key factor in the pathogenesis of SLE skin lesions, and apoptosis plays a critical role in regulating excessive cell growth, we further explored the impact of GEM on fibroblast apoptosis. Flow cytometry analysis of apoptosis showed that GEM knockdown significantly enhanced the apoptosis of both CCD-1066 and F-2408 fibroblast cell lines (**Figures 9E, G**). These findings suggest that GEM silencing promotes fibroblast apoptosis, potentially as a mechanism to counteract abnormal fibroblast proliferation.

## 4 Discussion

Systemic lupus erythematosus (SLE) is a complex autoimmune disease that has long imposed a significant burden on patients’ health, with progress in treatment remaining relatively slow. While current therapies such as immunosuppressants and anti-inflammatory drugs can effectively control symptoms and slow disease progression (42), the incomplete understanding of the underlying pathogenic mechanisms of SLE limits the ability to achieve full remission or precise treatment (43, 44). Therefore, it is crucial to further explore the pathophysiology of SLE, particularly the key cells and molecules involved in immune and inflammatory responses. Recent studies have highlighted the pivotal role of fibroblasts in the pathological process of SLE. Fibroblasts are involved not only in fibrosis and immune modulation but also in shaping the immune microenvironment, which may contribute to the chronic and progressive nature of the disease. However, the specific role of fibroblasts in SLE pathogenesis remains insufficiently studied, underscoring the need for more in-depth research to elucidate their contributions. Despite the recognized potential of precision immunotherapy in the treatment of SLE, advancements in this area have been slow. Precision medicine aims to tailor therapeutic strategies based on individual variations, particularly in the immune system (45). However, due to the high heterogeneity in clinical presentation and pathogenesis among SLE patients, existing research and treatment approaches have yet to enable truly personalized management. In this context, single-cell sequencing technology offers a promising opportunity to accelerate the progress of precision medicine. By performing detailed analyses at the single-cell level, researchers can uncover cellular and molecular alterations in SLE patients, identifying specific cell populations and biomarkers associated with disease progression. This technological advancement not only provides new insights for early diagnosis of SLE but also lays a solid foundation for developing personalized treatment strategies. Consequently, future research should focus on leveraging single-cell sequencing to further investigate the pathophysiological mechanisms of SLE, particularly the role of fibroblasts in immune modulation, and to promote the implementation of precision immunotherapy in SLE management.

This study provides new insights into the role of fibroblast subtypes in the pathogenesis of systemic lupus erythematosus (SLE), shedding light on the functional characteristics, differentiation potential, and regulatory mechanisms of these cells in disease contexts. Our results show that the C2 subtype, marked by the expression of GEM, plays a particularly prominent role in SLE. Fibroblasts in the SLE group were predominantly concentrated within the C2 subtype, highlighting its relevance in disease progression. Moreover, the elevated expression of pro-inflammatory genes and its significant involvement in the differentiation process of fibroblasts further underscores the importance of C2 in SLE pathology (46, 47).

The finding that fibroblasts in the SLE group were enriched in the C2 subtype is consistent with previous studies that have linked fibroblasts to autoimmune diseases and chronic inflammation (48, 49). Specifically, the C2 subtype demonstrated a higher expression of pro-inflammatory markers, such as IL16 and SIRT1, indicating its involvement in immune regulation (50). This aligns with earlier research suggesting that fibroblasts contribute to inflammation through the secretion of pro-inflammatory cytokines, which play a pivotal role in sustaining the chronic inflammatory environment characteristic of SLE. Furthermore, our pseudotime analysis suggests that the C2 subtype predominantly localizes at the later stages of differentiation, which corresponds to disease progression. This finding is significant because it suggests that the C2 subtype may not only be involved in the early immune responses but also contribute to the later, more chronic phases of SLE.

In comparison with previous studies, our findings further elucidate the association of the C2 fibroblast subtype with enhanced stemness and differentiation potential, thereby advancing our understanding of fibroblast plasticity in autoimmune diseases. Notably, C2 fibroblasts exhibited significantly elevated expression of stemness-associated genes, including MYC, TWIST, and HIF1A, indicating a more differentiated and functionally active population (51). These results align with prior research demonstrating that fibroblasts in chronic diseases frequently exhibit a “fibrotic” phenotype, actively contributing to tissue remodeling. Additionally, CytoTRACE scores and gene mining analyses reinforced the notion that C2 fibroblasts possess the highest differentiation potential, underscoring their critical role in disease progression. These findings highlight the therapeutic significance of modulating fibroblast differentiation as a potential strategy in systemic lupus erythematosus (SLE).

With regard to intercellular communication, our CellChat analysis provided a comprehensive mapping of the signaling networks governing fibroblast interactions within the SLE microenvironment. Specifically, the upregulation of CXCL and VEGF signaling pathways in C2 fibroblasts suggests a pivotal role in promoting endothelial cell migration and angiogenesis—biological processes that are frequently dysregulated in SLE (52). These observations are in concordance with previous studies reporting that fibroblasts actively contribute to vascular remodeling and inflammation in autoimmune conditions. Moreover, the pronounced responsiveness of C2 fibroblasts to incoming IFN-II and VCAM signals underscores the dynamic interplay between fibroblasts and immune cells in SLE pathogenesis.

A key discovery of this study is the identification of GEM as a defining marker of the C2 fibroblast subtype and a potential therapeutic target. Functional validation experiments demonstrated that GEM knockdown in fibroblast cell lines significantly impaired their proliferative, migratory, and survival capacities, suggesting a critical role for GEM in driving fibroblast dysfunction in SLE. Given the well-established contribution of fibroblasts to tissue fibrosis and inflammation in SLE skin lesions, the suppression of GEM expression not only attenuated fibroblast activity but also promoted apoptosis, thereby offering a potential mechanism to mitigate fibroblast-mediated pathology. These findings provide compelling evidence supporting the therapeutic potential of targeting GEM in SLE and other autoimmune diseases.

Despite these significant findings, our study has certain limitations. First, while we delineated the role of fibroblast subtypes in SLE, the precise mechanisms through which fibroblasts modulate immune responses remain incompletely understood and warrant further investigation. Additionally, our gene knockdown experiments utilized fibroblast cell lines, which may not fully recapitulate the complex *in vivo* microenvironment of SLE. Future studies employing primary patient-derived fibroblasts and *in vivo* models are necessary to validate these findings. Furthermore, while single-cell RNA sequencing (scRNA-seq) provided valuable insights into fibroblast heterogeneity, the integration of spatial transcriptomics would enable a more comprehensive understanding of fibroblast interactions within SLE lesions.

From a clinical perspective, our findings suggest that targeting specific fibroblast subtypes, particularly the C2 population, could yield novel therapeutic strategies for SLE management. Given the association of C2 fibroblasts with pro-inflammatory signaling, interventions aimed at inhibiting C2-related pathways, such as CXCL and VEGF signaling, may help modulate the inflammatory milieu in SLE. Additionally, therapeutic strategies targeting GEM expression or its downstream effectors may offer a promising avenue for controlling fibroblast activity and attenuating tissue fibrosis, a hallmark feature of SLE pathology.

Future research should focus on delineating the precise molecular mechanisms underlying fibroblast-mediated immune dysregulation in SLE. Investigating the transcriptional regulatory networks and epigenetic modifications that govern fibroblast differentiation and function will be essential for the development of targeted therapies. Furthermore, elucidating the crosstalk between fibroblasts and immune cells, including T cells and macrophages, could provide deeper insights into their role in disease pathogenesis.

In conclusion, this study offers critical insights into the role of fibroblast subtypes in SLE, particularly highlighting the pathogenic significance of the C2 subtype. The identification of GEM as a key regulator of fibroblast activity presents novel therapeutic opportunities for targeting fibroblast dysfunction in autoimmune diseases. Collectively, these findings contribute to a more comprehensive understanding of SLE pathogenesis and underscore the potential of fibroblast-directed therapies in disease management.

## 5 Conclusions

In this study, we identified and characterized distinct fibroblast subtypes associated with systemic lupus erythematosus (SLE), demonstrating the prominence of the C2 subtype in SLE pathology. Our findings indicate that the C2 fibroblast subtype exhibits elevated expression of pro-inflammatory genes, greater stemness potential, and a pronounced differentiation capacity, suggesting its critical role in disease progression. Functional analysis revealed that the C2 subtype is involved in key signaling pathways such as CXCL and VEGF, which may contribute to inflammatory and vascular remodeling processes in SLE. Furthermore, GEM gene knockdown significantly inhibited fibroblast proliferation and migration, highlighting its potential as a therapeutic target for regulating fibroblast activity in SLE. These results provide new insights into the fibroblast-mediated mechanisms underlying SLE and offer promising directions for future therapeutic interventions aimed at modulating fibroblast function in autoimmune diseases.

## Data Availability

The original contributions presented in the study are included in the article/**Supplementary Material**. Further inquiries can be directed to the corresponding author/s.

## Supplementary material

Supplementary Figure 1 GEM gene transfection knock-down low efficiency verification. Compared with untransfected cells, the mRNA level of CRYAB gene was significantly decreased in the transfected knockdown group.
