# Supplementary figures and images for: Investigation of the role of GEM in systemic lupus erythematosus through multi-omics joint analysis
Source: Front Immunol. 2025 Apr 9;16:1569605. doi: 10.3389/fimmu.2025.1569605 (PMC12014628; doi:10.3389/fimmu.2025.1569605)

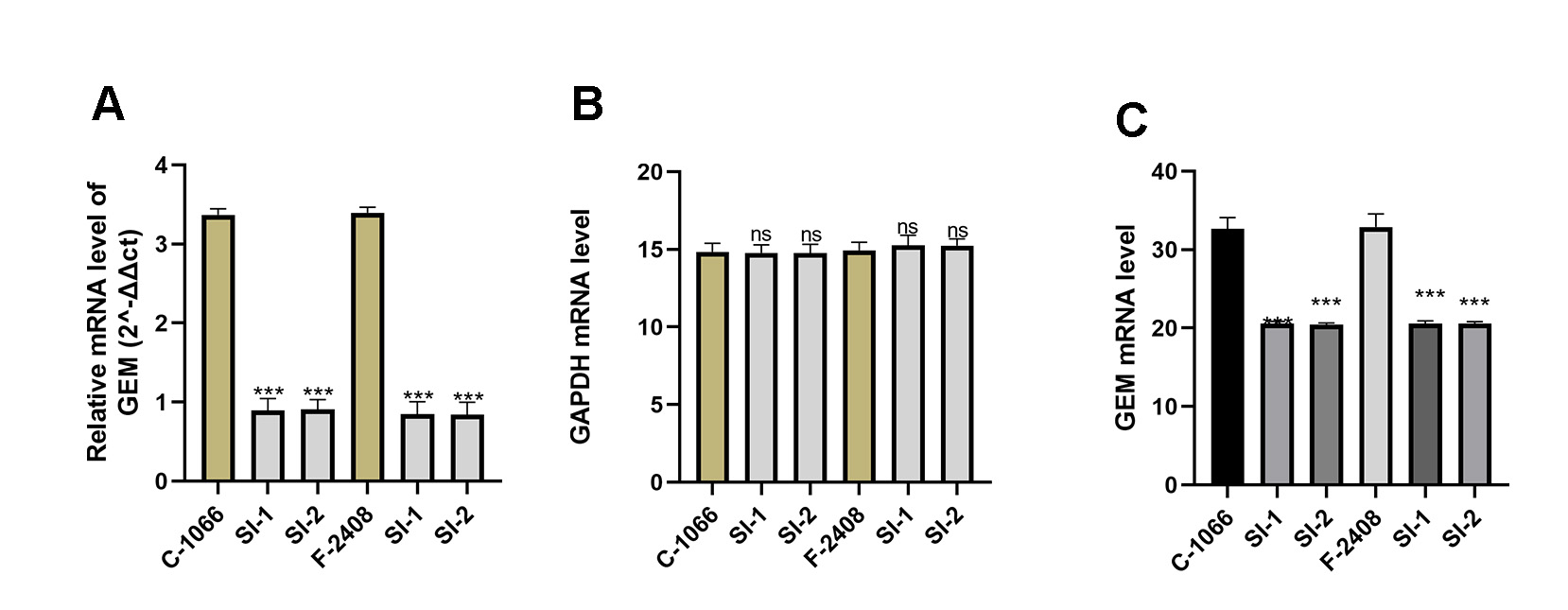

Supplement: Supplementary Figure 1 — GEM gene transfection knock-down low efficiency verification. Compared with untransfected cells, the mRNA level of CRYAB gene was significantly decreased in the transfected knockdown group. [file Image1.jpeg]
